# The Fermentation Process Improves the Nutritional Value of Rapeseed Cake for Turkeys—Effects on Performance, Gut Bacterial Population and Its Fermentative Activity

**DOI:** 10.3390/ani10091711

**Published:** 2020-09-22

**Authors:** Aleksandra Alicja Drażbo, Jerzy Juśkiewicz, Agata Józefiak, Paweł Konieczka

**Affiliations:** 1 Department of Poultry Science, Faculty of Animal Bioengineering, University of Warmia and Mazury in Olsztyn, Oczapowskiego 5, 10-719 Olsztyn, Poland; pawel.konieczka@uwm.edu.pl; 2 Institute of Animal Reproduction and Food Research of the Polish Academy of Sciences, Tuwima 10, 10-748 Olsztyn, Poland; j.juskiewicz@pan.olsztyn.pl; 3 Department of Preclinical Sciences and Infectious Diseases, Poznan University of Life Sciences, Wolynska 35, 60-637 Poznan, Poland; agata.jozefiak@up.poznan.pl

**Keywords:** gastrointestinal tract, microbiome, turkey, rapeseed cake, fermentation

## Abstract

**Simple Summary:**

Rapeseed cake (RC) could be valuable raw material in turkey diets, but its wide use is limited by the presence of anti-nutritional factors that are detrimental to gut function. The fermentation process contributes to the degradation of over 80% of carbohydrates, 30% of lignin, and 45% of total glucosinolates in RC, which are harmful to birds. Our research showed that fermentation can improve the nutritional value of RC, enabling good performance and maintainenance of a healthy gut in birds. Therefore, FRC appears to be highly promising in commercial turkey nutrition.

**Abstract:**

This experiment investigated the potential inclusion of fermented rapeseed cake (FRC) in turkey diets. The turkeys received diets either not supplemented (C) or supplemented with raw rapeseed cake (RRC) or FRC at 150 g/kg diet. In comparison with RRC, turkeys receiving FRC achieved significantly higher final BW comparable with that noted in the control group. The dietary inclusion of FRC increased the concentrations of propionic and valeric acid in the cecal digesta compared with the control group, and increased the proportion of butyric acid in SCFA profile compared with RRC group. The activities of glycolytic bacterial enzymes in the cecal digesta, were lowest in turkeys fed FRC. Experimental diets did not cause a shift in the relative abundances of the main bacterial phyla or orders in the cecal digesta. FRC increased the abundance of *Bacteroidaceae* at the family level, but decreased the abundance of *Lactobacillus* at the genus level compared with birds fed RRC. In conclusion, the dietary inclusion of FRC at 150 g/kg did not compromise bird performance, did not excessively stimulate bacterial activity, and did not cause shifts in the bacterial composition in the cecum. Actually, FCR exerted several beneficial effects that contributed to maintaining gut health in turkeys, which points to its advantage over RRC.

## 1. Introduction

Rapeseed cake (RC), a by-product of oil production, could be a valuable component of poultry diets because it contains up to 400 g/kg crude protein (CP) and high concentrations of metabolizable energy [1,2]. Unfortunately, wide use of RC is limited by the presence of non-starch polysaccharides (NSP) and other anti-nutritional factors such as glucosinolates, phytic acid, sinapine, and tannins [3]. High NSP levels in poultry diets may stimulate the proliferation and activity of gut microbiota [4], and increase the viscosity of small intestinal digesta, thus impairing the rate of food passage and nutrient utilization [3]. Glucosinolates hydrolyzed by the enzyme myrosinase are the most toxic compounds in rapeseed, and their maximum level in animal feed should not exceed 2.5 µmol/g [5]. Previous studies indicate that the impact of rapeseed products, rich not only in NSP, lignin and polyphenols but also in glucosinolates, may be more pronounced in turkeys than in broiler chickens due to a much longer fattening period in the former [4]. According to many authors, the inclusion rates of rapeseed products in poultry diets should not exceed 15–20% to prevent metabolic disorders caused by glucosinolates and not to compromise the growth performance of birds [6,7].

Previous research shows that fermentation can improve the nutritional value of diets by increasing protein availability and reducing the content of undesirable compounds in feed ingredients [7,8,9]. According to Rozan et al. [10], fermentation contributes to the degradation of 84% of carbohydrates, 30% of lignin, and 47% of total glucosinolates in rapeseed meal. Fermented feed components are usually characterized by higher counts of lactic acid bacteria, higher concentrations of selected volatile fatty acids, and a lower pH compared with raw materials, which can inhibit the growth of bacteria such as *Salmonella Typhimurium* and *Escherichia coli* in chickens [11]. Fermented feeds also contribute to increasing the abundance of beneficial microorganisms that exert probiotic effects in the gastrointestinal tract (GIT) [12]. Lactic acid and other short-chain fatty acids (SCFAs) are also produced by the indigenous microflora in the GIT, and are believed to play a role in reducing the numbers of many pathogens [13]. Lactic acid and acetic acid act synergistically against yeasts, molds and bacteria such as *Clostridium* and *Salmonella*. Propionic acid and butyric acid are also known as inhibitors of *Salmonella* growth [14]. Therefore, SCFAs, the major end products of fermentation, stabilize gut microbial composition and function, and constitute an additional energy source that can slightly improve the growth performance of birds [4].

The intestinal microbiota has recently been shown to largely affect host health and growth parameters through various functional roles in terms of nutrition and physiological metabolism [15]. Microbial diversity exerts a considerable effect on fermentation processes in the GIT. In poultry, the GIT harbors a very diverse microbial population, with over 600 different bacterial species representing more than 100 bacterial genera [16]. Unfortunately, the relevant data for turkeys are scarce, although the GIT is assumed to play a critical role in overall health in poultry.

The results of previous studies revealed that fermented raw material may have a positive influence on the gut ecosystem and morphology, immune function, and growth performance of birds [17]. However, the effect of fermentation of rapeseed products on gut function in turkeys remains insufficiently investigated. In view of the above, the objective of this study was to determine the effect of raw and fermented rapeseed cake on the physiological response of the GIT in turkey.

## 2. Materials and Methods

The study protocol was approved by the Local Ethics Committee for Animal Experiments in Olsztyn, Poland (decision number 30/2015). The animals were maintained accordingly to the guidelines comparable to EU Directive 2010/63/EU [18].

### 2.1. Rapeseed Cake

Rapeseed cake was purchased on the domestic market, from the Bielmar Fat and Oil Processing Plant in Bielsko-Biała, Poland. The raw material was ground and thoroughly mixed with water in a ratio of 1:2 in plastic containers. Rapeseed cake was fermented with the use of a commercial enzyme preparation of 6-phytase expressed in Pichia pastoris. The substrate was inoculated with enzymes (0.1% on a RC weight basis) and mixed. Solid-state fermentation was conducted for 24 h at 30 °C under anaerobic conditions. The enzymes were deactivated at 70 °C within 15 min, and the fermented biomass was dried at 55 °C. The fermentation process was carried out under patent-pending procedure No. 422849.

### 2.2. Animals and Experimental Design

The experimental material consisted of one-day-old female Hybrid Converter turkeys raised until 112 days of age. Turkeys were raised in pens on litter in a building with a controlled environment. Every pen was equipped with an automatic feeder and a bell-type drinker, and both water and feed were provided ad libitum. The temperature and lighting programs were consistent with the recommendations of Hybrid Turkeys [19].

The experiment was carried out on 1350 turkeys assigned to three experimental groups of 450 birds each (9 replications of 50 individuals each), which differed in the source of feed protein. In the control group, soybean meal was the main source of dietary protein, whereas the experimental groups were fed diets containing 15% of RRC or FRC. All diets were isocaloric and isonitrogenous, and contained similar amounts of major amino acids (including lysine, methionine with cysteine and threonine), minerals (including calcium and available phosphorus), and vitamins. The nutritional value of diets was consistent with the nutrient requirements of turkeys [19]. The compositions of control and experimental diets prepared in successive 4-week feeding periods are shown in Table 1.

The diets for the first period (days 1 to 28) were offered in crumble form, and the diets offered in the subsequent periods (days 29–112) were fed in pelleted form. At the end of each 4-week period, the body weight (BW) of turkeys, feed intake (FI) and mortality rates were recorded, and each pen of 50 birds was considered as an experimental unit. Feed conversion ratio (FCR) was calculated for each group based on BW gain and feed consumption.

### 2.3. Sample Collection

After 112 days of feeding, nine birds representing the average BW of each group were slaughtered by cervical dislocation to collect the test material. The ceacums were removed, emptied, and digesta was homogenized. Subsequently, samples were collected and used for the analysis of SCFAs and bacterial enzymes activity. The remaining portion of cecal digesta was transferred to test tubes and stored at −70 °C until needed.

### 2.4. Chemical Analysis

For chemical analyses, samples of RC were ground to pass through a 0.5-mm sieve. The samples were analyzed in duplicate for the content of DM, CP, and crude fiber (CF) according to AOAC [21] methods 934.01, 976.05 and 978.10, respectively. Gross energy (GE) was determined with an adiabatic bomb calorimeter (KL 12 Mn, Precyzja-Bit PPHU, Poland) standardized with benzoic acid. Phytate-phosphorus was determined as described by Haugh and Lantzsch [22]. Non-starch polysaccharides (NSP) were determined by gas-liquid chromatography (constituent neutral sugars) using an SP-2340 column and a Varian CP3380 gas chromatograph (Varian Inc., Palo Alto, CA, USA), and by colorimetry (uronic acids) using a Biochrom Ultrospec 50 (Biochrom Ltd., Cambridge, UK), according to the procedure described by Englyst and Cummings (1984, 1988) with modifications [23]. Uronic acids were determined as described by Scott [24]. Sugars (glucose—GLU, fructose, sucrose, raffinose, and stachyose) were determined as described by Slominski et al. [25]. Glucosinolates were determined by gas-liquid chromatography as described by Slominski and Campbell [26].

SCFAs were analyzed by gas chromatography (Shimadzu GC-2010, Kyoto, Japan) equipped with a capillary column (SGE, BP21, 30 m × 0.53 mm, SGE Europe Ltd., Kiln Farm Milton Keynes, UK). Samples of digesta (0.2 g) were mixed with 0.2 mL of formic acid, diluted with deionized water and centrifuged at 7211 g for 10 min. The supernatant was loaded onto a capillary column (SGEBP21, 30 m × 0.53 mm) using an on-column injector.

The activities of bacterial enzymes (α- and β-glucosidase, α- and β-galactosidase, β-glucuronidase, α-arabinopyranosidase, α-arabinofuranosidase β-xylosidase, β-cellobiosidase) released into the cecal environment were measured as the rate of p- or o-nitrophenol release from their respective nitrophenylglucosides [27]. The reaction mixture contained 0.3 mL of a substrate solution (5 mM) and 0.2 mL of a 1:10 (v/v) dilution of the cecal digesta sample in 100 mM phosphate buffer (pH 7.0) after centrifugation at 7211 g for 15 min. Incubation was carried out at 39 °C, and p-nitrophenol was quantified at 400 nm and at 420 nm (o-nitrophenol concentration) after the addition of 2.5 mL of 0.25 M cold sodium carbonate. Enzyme activity was expressed as μmol of the product formed per h per g of digesta.

### 2.5. Bacterial DNA Eextraction and 16SrRNA Sequencing

DNA was extracted from the cecal digesta with a commercial kit (Sherlock AX, A&A Biotechnology, Poland) according to the manufacturer’s instructions. The samples were mechanically lysed on FastPrep-24 on Zirconia beads (A&A Biotechnology, Poland) followed by additional lysis with enzymatic mix. The presence of bacterial DNA in the samples was confirmed using Real-Time PCR on the Mx3000P thermocycler (Stratagene, La Jolla, CA, USA) using SYBR Green as fluorochrome. Universal reaction primers, 1055F 5′-ATGGCTGTCGTCAGCT-3′ and 1392R 5′-ACGGGCGGTGTGTAC-3′, were used for the amplification of 16S rDNA. The temperature profile of the reaction was as follow: 95 °C, 3 min; 95 °C, 15 s; 58 °C, 30 s; 72 °C, 30 s; Tm 65 °C -> 95 °C. DNA was quantified using the NanoDrop (NanoDrop Technologies, Wilmington, DE, USA) and standardized at a final concentration of 5 ng/μL. Microbial diversity was determined by sequencing the amplified V3-V4 region of the 16S rRNA gene with the use of the following primers: 16S Amplicon PCR Forward Primer 5′TCGTCGGCAGCGTCAGATGTGTATAAGAGACAGCCTACGGGNGGCWGCG 16S Amplicon PCR Reverse Primer 5′GTCTCGTGGGCTCGGAGATGTGTATAAGAGACAGGACTACHVGGGTATCTAATC.

The following PCR conditions were applied: 95 °C for 3 min; 25 cycles of 95 °C for 30 s, 55 °C for 30 s, 72 °C for 30 s, 72 °C for 5 min, hold at 4 °C. The expected product size on a Bioanalyzer trace after the Amplicon PCR step was ~550 bp. The PCR products were cleaned up using AMPure XP beads. The libraries were sequenced running 2 × 250 bp paired-end reads. The PCR products were cleaned and the library was combined with the sequencing adapters and dual indices using the Nextera XT Index Kit (Illumina, San Diego, CA, USA) according to the 16S metagenomic sequencing library preparation instruction (Illumina, San Diego, CA, USA). The PCR with Nextera XT Index Primers was carried out under the following conditions: 95 °C for 3 min; 8 cycles of 95 °C for 30 s, 55 °C for 30 s, 72 °C for 30 s, 72 °C for 5 min, hold at 4 °C. The PCR products were cleaned up again with AMPure XP beads. The final library was validated to the expected size on a Bioanalyzer trace of ~630 bp. The libraries were quantified using a fluorometric quantification method and dsDNA binding dyes. The individual concentrations of DNA libraries were calculate in nM, based on the size of DNA amplicons as determined by the Agilent Technologies 2100 Bioanalyzer.

For sequencing the individual libraries, they were diluted to 4 nM, denatured with 10 mM Tris pH 8.5 and spiked with 20% (v/v) of PhiX. An aliquot of 5 μL of diluted DNA was mixed to prepare pooled libraries for the MiSeq (Illumina, San Diego, CA, USA) run. > 100,000 reads were performed per sample. The sequencing data were clustered into operational taxonomic units (OTUs), and were classified at several taxonomic levels, kingdom, phylum, class, order, family, genus and species. The Greengenes database was used for metagenomic analysis.

### 2.6. Statistical Analysis

For a statistical analysis of performance parameters, a single pen (*n* = 9) was considered as a replicate experimental unit. For analyses of the physiological and microbiological parameters of the GIT, individual birds were considered as experimental units. All analyses were performed on 27 birds representing 9 replicates from each of the 3 experimental groups. One-way analysis of variance (ANOVA) was performed with the use of Statistica 10.0 software (StatSoft, Krakow, Poland). When a significant treatment effect was noted, the post-hoc Tukey test was used to determine differences between treatment groups. Data were presented as means±SEM, and the value of *p* < 0.05 was considered statistically significant. The post-hoc Tukey HSD test did not reveal significant differences between group means with regard to the BW of birds and butyric acid, although *p*-value reached 0.04. Therefore, Duncan’s test was used, and the observed significant differences were considered as tendencies. The significance of differences between livability data was determined by the Kruskal-Wallis test by ranks (non-parametric ANOVA on ranks).

## 3. Results

Fermentation increased the content of DM, CP, and CF, and decreased the content of anti-nutritional factors in RC (Table 2). In comparison with RRC, FRC had nearly ten-fold lower glucosinolates content, nearly two-fold lower carbohydrates content and nearly twenty-fold lower content of phytate-phosphorus. Fermentation had no effect on NSP concentrations in RC.

### 3.1. Effect of Diets Containing RRC or FRC on Growth Performance.

The inclusion of 15% of RRC or FRC in turkey diets had no influence on the growth performance parameters of birds, including DFI, FCR, and livability (Table 3). However, the average final BW of turkeys receiving RRC tended to decrease in comparison with the remaining groups. Fermentation increased the final BW of turkeys, which was approximately 1.3% higher in the FRC group than in the RRC group, and comparable with that in the control group (*p* = 0.043).

### 3.2. Effect of Diets Containing RRC or FRC on SCFA Concentrations and the Activities of Bacterial Enzymes in the Cecal Digesta

The inclusion of RRC or FRC in turkey diets slightly affected SCFA concentrations in the cecal digesta (Table 4). Neither total SCFA concentrations nor the total pool or the total concentrations of putrefactive SCFAs differed between the treatments (*p* > 0.05). In the SCFA profile, acetic acid predominated, followed by butyric acid and propionic acid. The proportion of butyric acid in the SCFA profile was higher in birds fed FRC than in those fed RRC (*p* = 0.047). The concentrations of propionic acid and valeric acid were higher in FRC-fed birds compared with the control treatment (*p* = 0.028 and *p* = 0.048, respectively).

An analysis of the activities of bacterial enzymes in the cecal digesta (Table 4) indicated that only α-glucosidase and β-galactosidase were not affected by the dietary treatments (*p* > 0.05). More specifically, the activities of β-glucosidase (*p* = 0.026), α-galactosidase (*p* = 0.024), α-arabinopyranosidase (*p* < 0.001), α-arabinofuranosidase (*p* < 0.001), β-xylosidase (*p* < 0.001), and β-celobiosidase (*p* < 0.001) were lower in birds fed diets with FRC vs. RRC. In comparison with the control treatment, the administration of RRC or FRC resulted in lower activity of β-glucuronidase in the cecal digesta (*p* < 0.001).

### 3.3. Effect of Diets Containing RRC or FRC on the Microbial Composition in the Cecal Digesta

A metagenomic analysis of the cecal digesta revealed that the predominant bacterial phyla were *Firmicutes, Bacteroidetes, and Proteobacteria* (Table 5). Their relative abundances were as follows: *Firmicutes*—around 50% (49.42201003350.33%), *Bacteroidetes*—around 20% (19.15–28.64%), *Proteobacteria*—around 5% (4.68–5.33%), and *Cyanobacteria*—around 2.5% (1.35–3.22%) of OTUs. Unclassified bacterial 16SrRNA sequences accounted for 6.44 to 7.04%. The inclusion of RRC or FRC in turkey diets did not affect the bacterial populations (OTUs) at the phylum or order levels. The dietary treatments had a minor effect on the relative abundances of bacteria at the family and genus levels. Fermented rapeseed cake caused a significant increase in the relative abundance of *Bacteroidaceae* (*p* = 0.036 vs. C). The relative abundance of *Lactobacillus* decreased significantly in the FRC group, and increased in the RRC group (*p* = 0.004 vs. C).

## 4. Discussions

In the present study, RC fermentation marginally affected its chemical composition, including the content of CP, CF, and GE. These results corroborate our previous findings [28]. However, the fermentation process decreased the concentrations of anti-nutritional factors and indigestible substances including glucosinolates and phytate phosphorus, which are detrimental to poultry. Therefore, our data indicate that RC fermentation may be beneficial since the predictable value of raw materials after processing is one of the key determinants of their use as feedstuffs in turkey diets.

The present report demonstrated that diets containing 150 g/kg of FRC were well utilized by birds, as their growth performance was marginally affected in the experimental groups and remained within the limits typical of female hybrid turkeys of the same age [19]. The fermentation process was beneficial because RRC decreased the final BW of turkeys, relative to the control group, whereas this effect was not observed in birds fed FRC. These data are consistent with the findings of Rad-Spice et al. [29] who noted adverse effects of the dietary inclusion of raw rapeseed in the amount of 150 g/kg or higher on the performance of broiler chickens. Therefore, fermentation enables to supplement turkey diets with RC at 150 g/kg without compromising bird performance.

The nutritional value of different feedstuffs is largely determined by their influence on the physiological status of the gut. In the current study, diets containing either RRC or FRC had a significant effect on cecal parameters, as selected SCFAs concentration and activities of bacterial enzymes. Dietary substrates, including NSP and protein, which are not fully digested and absorbed in the upper GIT of birds, may accumulate in the distal part of the gut, thus altering the profile and activity of microbiota, which manifests as changes in SCFA concentrations. Short-chain fatty acids are an important energy source for the intestinal epithelium, and they can also regulate mucin production and intestinal immune responses, which play a key role in the development of microbiota and in the prevention of pathogen colonization [13,30]. In the cecum, which is the main site of fermentation in birds, the digesta is fermented for a longer period of time, and the concentrations of SCFAs are higher than in the ileum. The SCFAs produced in the cecum also play an important role in maintaining bird health [31]. In our study, diets containing RC did not induce excessive fermentation in the cecum since total SCFA concentrations were similar in turkeys fed experimental and control diets. Acetic, butyric and propionic acids are the most abundant SCFAs in the cecal digesta of birds fed different diets. In the current experiment, the above acids were also present in the highest concentrations in the cecal digesta of turkeys, which indicates that experimental diets containing RC did not disturb cecal fermentation [4]. Interestingly, our findings revealed that the replacement of RRC with FRC in turkey diets caused a beneficial shift in the concentration of propionic acid and in the proportion of butyric acid in the SCFA profile. The beneficial metabolic exerted by propionic acid is suggested to increase insulin sensitivity and glucose tolerance as sensed in the portal vein [32], whereas butyric acid is the preferred energy source for enterocytes, it exerts bacteriostatic effects on selected enteric bacteria, and may reduce the abundance of *Enterobacteriaceae* including *Salmonella* [14]. Furthermore, butyrate can also affect the hormonal and nervous systems, and therefore affect host physiology, including protection against colorectal cancer, inflammation, and appetite regulation [33]. Branched SCFAs such as isobutyrate and isovalerate are the end-products of protein fermentation, whereas acetic acid, propionic acid, and butyric acid are generated through the fermentation of dietary fibers [34,35]. Thus, in the light of our findings (a minor effect of RC on the SCFA profile and the concentrations of individual fatty acids), the inclusion of RRC and, in particular, FRC in turkey diets did not lead to an excess supply of fermentative protein and fiber substrates. Therefore, RC is unlikely to cause a negative shift in cecal fermentation. The above results indicate that FRC was well tolerated by birds. The present findings, unlike those reported by Mikulski et al. [6], confirm that rapeseed cultivars have been significantly improved in recent years. Therefore, rapeseed products can be included in turkey diets, especially that additional processing such as fermentation can further improve their nutritional value.

The activities of bacterial enzymes in the cecum are indicative of dietary ingredient-host interactions. The physicochemical structure of the substrates that escaped digestion in the upper GIT affects the activities of bacterial enzymes. In the current study, the use of FRC, but not RRC, downregulated microbial activity in the cecal ecosystem; the activities of most enzymes (in particular β-glucuronidase, α-arabinopyranosidase and α-arabinofuranosidase) were lower in birds fed diets with FRC than in those fed the control diet. The decreased activity of β-glucuronidase may be indicative of reduced *E. coli* and *Clostridium* populations, and a lower risk of glucuronide hydrolysis in the gut lumen, which generates toxic and carcinogenic substances from nontoxic glycosides [36]. Similarly, the decreased activities of α-arabinopyranosidase, α-arabinofuranosidase and β-xylosidase suggest that there was no shift in the abundance of residing bacteria, which can release arabinose, xylose, arabinose, and galactose from dietary substrates [37].

The microbiome of the intestinal tract plays a fundamental role in gut health by modulating the fermentation of polysaccharides and the metabolism of nitrogen, fatty acids, and lipids. The diversity of gastrointestinal microbiome is believed to have a critical effect on the overall health status of birds. Despite the fact that the interaction between gut microbiota and health status has not been fully elucidated to date, it can be used as a reliable indicator of birds’ response to dietary treatment. 16S rRNA next-generation sequencing enables to describe all detected microbial populations in one analysis. The results of metagenomic bacterial analyses do not depend on bacterial culture conditions and stand in opposition to direct DNA-DNA hybridization methods. The metagenomic data of 16S rRNA sequencing provide important information about the genetic material of the analyzed bacteria. In previous studies [38,39] the bacterial phyla of *Firmicutes* and *Bactroidates* were most abundant in the ceca of tukeys, which is consistent with our findings. In the present experiment, the above phyla accounted for 53.5% and 24.0%, respectively, of the cecal microbiota in turkeys. Moreover, the experimental diets had no significant influence on the relative abundances of the main phyla of bacteria residing in the ceca. However, they significantly affected the abundance of the family *Bacteroidaceae* in the ceca, which was greater in birds fed FRC than in those fed RRC, whereas the opposite was noted for the genus *Lactobacillus*. Since the abundance of *Bacteroidaceae* in birds fed FRC did not differ significantly from that noted in the control birds, their shift should not be attributed to the adverse effect of FRC inclusion. Regarding *Lactobacillus* bacteria, which are defined as microorganisms exerting a beneficial effect on the host, their significantly lower abundance in turkeys fed FRC than in those fed RRC could be considered undesirable. According the Józefiak et al. [40], an increase in the counts of *Bacteroides* and a decrease in the counts of *Lactobacilli* are negatively correlated with intestinal health. However, it should be noted that in the present study, *Lactobacillus* abundance was similar in turkeys fed FRC and in control birds, thus suggesting that the experimental diets were unlikely to exert an adverse effect on gut microbiota by causing a shift in the abundance of bacteria residing in the cecum. The significantly higher abundance of *Lactobacillus* in birds fed RRC compared with the remaining treatments, could result from the fact that more substrates escaped digestion in the upper GIT of turkeys and were able to enter their ceca [41], thus supporting *Lactobacillus* proliferation. This speculation was also supported by the fact that the performance of birds fed FRC was not compromised relative to the RRC group, indicating that the reduction in *Lactobacillus* abundance in the FRC treatment was marginal in terms of the gut health status. These findings, as well as the responses of SCFAs and bacterial enzymes reported above, appear to confirm that FRC exerted a negligible but generally beneficial effect on gut physiology in turkeys.

## 5. Conclusions

The results of this study indicate that FRC can be included in turkey diets at 150 g/kg without compromising the growth performance of turkeys and maintaining a healthy gut. Our findings also suggest that in comparison with unprocessed RC, the fermentation process improved the nutritional value of RC and exerted beneficial effects on SCFA concentrations and the activities of bacterial glycolytic enzymes. Therefore, fermentation represents an interesting approach to increasing the efficacy of RC in commercial turkey nutrition.

## Figures and Tables

**Table 1:** Composition and calculated analysis of control (C) and experimental diets containing raw rapeseed cake (RRC) or fermented rapeseed cake (FRC) fed during four feeding periods to turkeys aged 1–112 days, in g/kg as-fed basis (unless indicated otherwise).

Compounds	Weeks 1 to 4			Weeks 5 to 8			Weeks 9 to 12			Weeks 13 to 16		
	C	RRC	FRC	C	RRC	FRC	C	RRC	FRC	C	RRC	FRC
Wheat	519.4	429.3	429.3	522.2	434.6	434.6	627.3	539.7	539.7	721.4	633.7	633.7
Soybean meal	414.8	345.5	345.5	388.5	318	318	282.4	211.9	211.9	201	130.5	130.5
Rapeseed cake	—	150.0	—	—	150.0	—	—	150.0	—	—	150.0	—
Fermented rapeseed cake	—	—	150.0	—	—	150.0	—	—	150.0	—	—	150.0
Soybean oil	16.6	28.3	28.3	40.9	52.1	52.1	49.5	60.8	60.8	47.4	58.7	58.7
Monocalcium phosphate	17.8	17	17	15.5	15	15	11.4	10.9	10.9	7	6.5	6.5
Sodium bicarbonate	1.9	1.9	1.9	1.5	1.5	1.5	1.5	1.5	1.5	1.5	1.5	1.5
Sodium chloride	2	1.8	1.8	1.8	1.8	1.8	1.6	1.6	1.6	1.2	1.2	1.2
Limestone	15.2	14	14	16.8	15.3	15.3	14.3	12.8	12.8	10.3	8.8	8.8
Choline chloride	0.9	0.9	0.9	1	1	1	1	1	1	1	1	1
L-Lysine HCL	4.3	4.7	4.7	4.6	4.6	4.6	4.6	4.5	4.5	3.7	3.6	3.6
DL-Methionine	3.3	2.9	2.9	3	2.4	2.4	2.3	1.7	1.7	1.9	1.3	1.3
L-Threonine	1.1	1	1	1.3	0.9	0.9	1.3	0.9	0.9	0.9	0.5	0.5
Enzymes	0.3	0.3	0.3	0.3	0.3	0.3	0.3	0.3	0.3	0.3	0.3	0.3
Vitamin-mineral premix ^1^	2.5	2.5	2.5	2.5	2.5	2.5	2.5	2.5	2.5	2.5	2.5	2.5
Analyzed nutrients												
Crude protein	271	277	277	258	255	255	220	226	226	184	186	186
Crude fat	40.5	59	59	52.6	75.6	75.6	54.3	69.9	69.9	60.3	75.1	75.1
Calculated nutritional value ^2^												
ME, kcal/kg	2750	2750	2750	2900	2900	2900	3050	3050	3050	3125	3125	3125
Crude fiber	26.9	45.5	45.5	28.2	46.5	46.5	26.7	45	45	25.9	44.2	44.2
Lysine	17.4	17.4	17.4	16.3	16.3	16.3	13.6	13.6	13.6	10.9	10.9	10.9
Arginine	17.4	17.4	17.4	15.8	15.6	15.6	12.8	12.6	12.6	10.5	10.4	10.4
Methionine	6.8	6.7	6.7	6.4	6	6	5.2	4.8	4.8	4.4	4.1	4.1
Methionine + Cysteine	11.3	11.3	11.3	10.5	10.5	10.5	8.8	8.8	8.8	7.7	7.7	7.7
Threonine	10.4	10.4	10.4	10	10	10	8.4	8.4	8.4	6.8	6.8	6.8
Tryptophan	3.4	3.4	3.4	3.1	3.1	3.1	2.6	2.6	2.6	2.2	2.3	2.3
Ca	13	13	13	11	11	11	9	9	9	6.5	6.5	6.5
Available P	7	7	7	5	5	5	4	4	4	3	3	3

^1^ provided the following per kilogram of diet in weeks 1–8 and 9–16: vitamin A, 12,500 and 9600 IU; vitamin D3, 5000 and 4800 IU; vitamin E, 100 and 60 mg; vitamin K3, 4 and 3 mg; vitamin B1, 4.5 and 2 mg; vitamin B2, 15 and 12 mg; vitamin B6, 5 and 5 mg; vitamin B12, 16 and 0.03 mg; folic acid, 3 and 2.5 mg; pantothenic acid, 28 and 23 mg, nicotinic acid, 110 and 85 mg; biotin, 0.38 and 0.38 mg; Mn, 160 and 120 mg; Zn, 160 and 120 mg; Fe, 80 and 40 mg; Cu, 25 and 25 mg; I, 2.5 and 2 mg; Se, 0.3 and 0.3 mg, respectively ^2^ Calculated according to Polish Feedstuff Analysis Tables [20].

**Table 2:** Chemical composition of raw rapeseed cake (RRC) and fermented rapeseed cake (FRC) (g/kg, dry matter basis).

Component	RRC	FRC
Dry matter	912	935
Crude protein	325	349
Crude fiber	155	164
Gross energy (kcal/kg)	5127	5240
Phytate-phosphorus	3.07	0.16
NSP ^1^	222	226
Glucosinolates ^2^ (µmol/g)	16.3	1.66
Sugars ^3^	92.2	54.8

^1^ non-starch polysaccharides including rhamnose, arabinose, xylose, mannose, galactose, glucose, uronic acids; ^2^ including gluconapin, glucobrassicinapin, progoitrin, glucobrassicin, hydroxyglucobrassicin; ^3^ including glucose, fructose, sucrose, raffinose, and stachyose.

**Table 3:** Growth performance of turkeys fed a control diet (C) and diets containing raw rapeseed cake (RRC) or fermented rapeseed cake (FRC).

Item	Dietary Treatment			SEM	*p*-Value
	C	RRC	FRC		
DFI, g	258	256	261	1.372	0.336
BW, kg	10.82 ^x^	10.68 ^y^	10.83 ^x^	0.027	0.043
FCR, kg/kg	2.53	2.51	2.52	0.010	0.819
Livability, %	99.1	99.8	97.6	0.381	0.165

^x,y^ means within a row with different superscripts were considered as a near-significant trend; DFI—daily feed intake; BW—body weight; FCR—feed conversion ratio.

**Table 4:** Concentrations of short-chain fatty acids (SCFAs) and activities of selected bacterial enzymes in the cecal digesta of turkeys fed a control diet (C) and diets containing raw rapeseed cake (RRC) or fermented rapeseed cake (FRC) from 1 to 112 days of age ^1^.

Item	Dietary Treatment			SEM	*p*-Value
	C	RRC	FRC		
SCFA concentrations (μmol/g)					
Acetic acid	96.4	100	98.3	2.129	0.763
Propionic acid	7.44 ^b^	8.70 ^ab^	11.2 ^a^	0.599	0.028
Iso-butyric acid	0.949	1.07	1.02	0.055	0.671
Butyric acid	21.4	21.4	26.6	1.103	0.082
Iso-valeric acid	1.28	1.31	1.41	0.065	0.723
Valeric acid	1.37 ^b^	1.65 ^ab^	2.00 ^a^	0.106	0.048
Total SCFAs	129	134	141	3.235	0.351
SCFA profile (% of total SCFAs)					
Acetic acid	74.8 ^a^	74.7 ^a^	70.2 ^b^	0.678	0.003
Propionic acid	5.88	6.45	7.8	0.362	0.08
Butyric acid	16.4 ^xy^	15.8 ^y^	18.8 ^x^	0.536	0.047
Total pool of SCFAs (μmol/kg BW)	360.0	333.0	451.0	28.40	0.215
Total putrefactive SCFAs	3.60	4.03	4.43	0.151	0.079
Enzyme activity (U/g)					
α-glucosidase	28.6	23.2	31.5	1.759	0.154
β-glucosidase	1.32 ^ab^	1.39 ^a^	0.933 ^b^	0.076	0.026
α-galactosidase	14.6 ^ab^	19.6 ^a^	12.8 ^b^	1.093	0.024
β-galactosidase	33.2	32.6	27.4	1.344	0.161
β-glucuronidase	35.5 ^a^	17.4 ^b^	8.23 ^b^	2.937	0.001
α-arabinopyranosidase	2.04 ^a^	2.36 ^a^	1.32 ^b^	0.130	0.001
α-arabinofuranosidase	3.71 ^a^	4.64 ^a^	1.95 ^b^	0.284	0.001
β-xylosidase	4.47 ^b^	13.1 ^a^	3.75 ^b^	1.166	0.001
β-celobiosidase	1.75 ^b^	2.65 ^a^	1.37 ^b^	0.140	0.001

^1^ data representing mean values of 9 birds per treatment; ^a,b^ means within a row with different superscripts differ significantly (*p* ≤ 0.05); ^x,y^ means within a row with different superscripts were considered as a near-significant trend.

**Table 5:** Relative abundance of bacterial communities in the cecal digesta of turkeys fed a control diet (C) and diets containing raw rapeseed cake (RRC) or fermented rapeseed cake (FRC) ^1^.

Item	Dietary Treatment			SEM	*p*-Value
	C	RRC	FRC		
Phylum					
*Firmicutes*	50.33	60.89	49.42	9.04	0.053
*Cyanobacteria*	1.35	2.83	3.22	1.97	0.204
*Actinobacteria*	10.39	4.97	5.62	5.56	0.167
*Proteobacteria*	5.33	4.68	4.73	1.62	0.711
*Bacteroidetes*	24.13	19.15	28.64	9.16	0.181
Order					
*Lactobacillales*	9.45	24.89	7.27	18.02	0.166
*Clostridiales*	36.63	31.91	36.80	10.64	0.629
*Bifidobacteriales*	9.75	4.39	4.98	5.40	0.155
*Bacteroidales*	16.54	12.42	20.51	6.10	0.071
Unclassified	12.77	12.71	13.46	1.86	0.711
*Nostocales*	2.13	2.55	3.36	1.72	0.649
*Sphingobacteriales*	4.97	4.87	4.85	1.90	0.993
*Flavobacteriales*	1.90	2.04	2.02	0.45	0.871
Family					
*Clostridiaceae*	15.11	13.32	13.63	6.53	0.862
Unclassified	20.24	19.08	20.52	2.94	0.631
*Lachnospiraceae*	7.09	6.42	9.21	3.43	0.322
*Lactobacillaceae*	9.09	29.52	6.30	18.61	0.117
*Bifidobacteriaceae*	12.66	5.19	9.33	5.88	0.188
*Ruminococcaceae*	9.49	8.70	8.88	3.59163	0.915
*Bacteroidaceae*	8.20 ^ab^	5.38 ^b^	11.42 ^a^	3.97	0.036
*Sphingobacteriaceae*	5.77	5.76	5.43	1.22	0.909
*Rikenellaceae*	6.23	6.19	5.85	1.67	0.920
Genus					
Unclassified	24.65	25.31	26.26	4.26	0.780
*Lactobacillus*	7.78 ^b^	37.31 ^a^	5.95 ^b^	11.60	0.004
*Clostridium*	4.61	4.38	5.68	1.92	0.521
*Bifidobacterium*	11.97	5.12	7.42	6.62	0.333
*Blautia*	6.60	5.45	7.74	3.07	0.427
*Alkaliphilus*	8.68	7.55	7.71	4.79	0.899
*Faecalibacterium*	7.41	6.09	6.08	3.44	0.780

^1^ data representing mean values of 9 birds per treatment; ^a,b^ means within a row with different superscripts differ significantly (*p* ≤ 0.05).
